# A Terahertz Optomechanical Detector Based on Metasurface and Bi-Material Micro-Cantilevers

**DOI:** 10.3390/mi13050805

**Published:** 2022-05-21

**Authors:** Hailiang Zhu, Kai Wang, Ganyu Liu, Gengchen Wang, Jinchao Mou, Weiwei Zhang, Gao Wei

**Affiliations:** 1 School of Electronics and Information, Northwestern Polytechnical University, Xi’an 710072, China; zhuhl@nwpu.edu.cn (H.Z.); liugy@mail.nwpu.edu.cn (G.L.); wanggc@mail.nwpu.edu.cn (G.W.); weigao@nwpu.edu.cn (G.W.); 2 Beijing Research Institute of Telemetry, Beijing 100097, China; jasongor2021@163.com (J.M.); zizhulin511@163.com (W.Z.)

**Keywords:** optomechanical detector, terahertz, metasurface absorber, bi-material micro-cantilever

## Abstract

Terahertz imaging technology has shown great potential in many fields. As the core component of terahertz imaging systems, terahertz detectors have received extensive attention. In this paper, a metasurface-based terahertz optomechanical detector is proposed, which is made of two fabrication-friendly materials: gold and silicon nitride. The optomechanical detector is essentially a thermal detector composed of metasurface absorber, bi-material micro-cantilevers and heat insulation pillars. Compared with traditional thermal terahertz detectors, the optomechanical detector employs a metasurface absorber as the terahertz radiation coupler and obtains an absorptivity higher than 90% from 3.24 to 3.98 THz, which is much higher than that of traditional terahertz detectors with absorbers made from natural materials. Furthermore, the detector is fabricated by MEMS process and its responsivity has been verified by a specifically designed optical read-out system; the measured optomechanical responsivity is 24.8 μm/μW, which agrees well with the multi-physics simulation. These results indicated that the detector can be employed as a pixel to form a terahertz focal plane array in the future, and further realize real-time terahertz imaging at room temperature.

## 1. Introduction

Terahertz waves are located between microwave and infrared light, referring to electromagnetic waves with frequencies from 0.1 to 10 THz [1]. Due to the distinctive characteristics, terahertz technologies have shown great potential in the fields of biological detection [2], high-speed communication [3] and security detection [4,5]. Among plenty research of terahertz technologies and devices, high sensitivity terahertz detectors, as the core components of terahertz imaging systems, have received extensive attention [6]. Terahertz detectors can be divided into two categories with different mechanisms: photon detectors and thermal detectors. The photon detectors utilize the optoelectronic properties of materials to characterize terahertz wave power in the form of photocurrent signals [7,8,9]. Conventional photon detectors include superconducting tunnel junction detectors [10,11], and photoconductive detectors [12,13], which can achieve high sensitivity and extremely fast response speed, but they need to work at ultra-low temperatures and have narrow operating bandwidths. The imaging systems with photon detectors have to equip additional cooling device for this purpose, which is unfriendly for practical application. As for thermal detectors, they convert incident terahertz waves into temperature change, and characterize the terahertz wave power by thermal structures such as thermistors [4,14]. Compared to photon detectors, the response time of thermal detectors has increased, but they can work at room temperature (293.15 K), or lower temperature to achieve higher sensitivity [15].

Imaging time plays an important role in terahertz imaging systems. Similar to THz-TDS, terahertz scanning imaging systems need to record each pixel by a photoconductive antenna in turn, and finally synthesize a complete two-dimensional image [16]. Depending on the resolution of image, this process may take tens of seconds or even several hours, which is unable to meet the demand for real-time terahertz imaging. In recent years, the concept of a terahertz focal plane array (FPA) imaging system has been proposed and has attracted much attention. The system images favour staring; that is, the terahertz signal of observed object at a certain moment is mapped onto the FPA, then each unit cell in the FPA responds independently and simultaneously according to the signal strength they have received [6,15]. The array response signals are read out by high-speed read-out systems, so that a complete two-dimensional image can be obtained within tens of milliseconds, which is much faster than the scanning imaging systems and suffices the demand of real-time imaging [17]. Several FPA imaging systems adopting microbolometers as their array unit cell have been demonstrated to have real-time imaging capability in terahertz band [4,15,18]. However, the lack of process compatible materials with high-absorptivity in terahertz band has reduced the responsivity of microbolometers, further negatively influencing the sensitivity and image quality of FPA imaging systems.

Introducing metasurface into terahertz detectors can greatly improve the absorptivity of signal coupling part [19]. Metasurfaces are artificial two-dimensional periodic structures composed of several subwavelength cells arranged in a planar array [20]. By optimizing the size, structure and periodicity of cells, the metasurface with different electromagnetic properties can be obtained, further realizing the regulation of amplitude and phase of the electromagnetic waves [21,22]. Metasurface absorbers for bi-material micro-cantilever terahertz detectors are required to obtain high absorptivity and significant photothermal responsivity to meet the demand of high-sensitivity detection [23,24]. Moreover, the efficient absorption band of metasurface is greatly affected by process errors, which will cause unpredictable shifts in the operating frequency of detectors.

The features mentioned above bring challenges to designing high-sensitivity, and real-time terahertz detectors that can operate at room-temperature. In this paper, we propose a terahertz optomechanical detector based on metasurface and bi-material micro-cantilevers. The absorptivity of metasurface is higher than 90% from 3.24 to 3.98 THz, approximately twice that of conventional film absorbers. Similar to the mechanism of the microbolometer, the metasurface absorber converts energy of incident terahertz waves into heat, and transfers heat to bi-material micro-cantilevers through their interfaces. Since the thermal expansion coefficients of two layers in bi-material micro-cantilevers are quite different, the free ends of micro-cantilevers will bend to the side with a smaller thermal expansion coefficient. The displacement of free ends can be read out by several methods and used to characterize the incident terahertz energy. Compared with traditional terahertz detectors, the optomechanical detector proposed in this paper can operate in a specialized frequency band with less response time and can also form big planar arrays to meet the requirements of FPA imaging systems, which is beyond the reach of the Golay cell, natural absorber-based bolometer and photoconductive antennas [25,26,27]. In this work, the displacement is measured by a specifically developed optical read-out system, which avoids the integration of complex read-out circuits and simplifies the design process while ensuring the correctness of test results. Simulations and experiments in this paper are carried out at room temperature (293.15 K) with one atmosphere, and the measured optomechanical responsivity of the detector is 24.8 μm/μW. The design and analysis of bi-material micro-cantilevers, metasurface absorber, the optomechanical detector and the measurement system based on optical read-out method will be detailed below.

## 2. Design and Simulation Discussions

**Figure 1:**
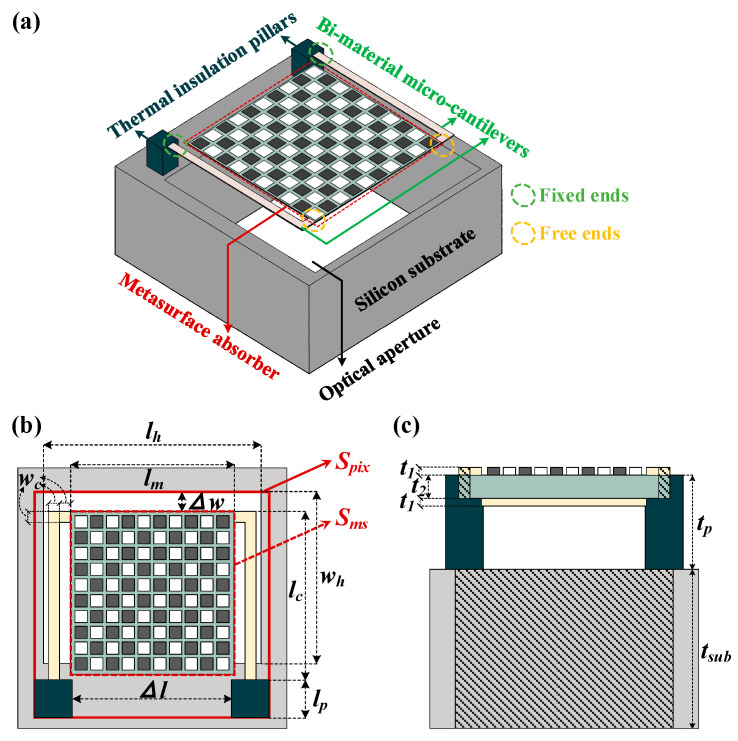
(**a**) 3D view of the detector with bi-material micro-cantilevers and metasurface absorber, fabricated on silicon substrate with optical aperture. (**b**) Top view and (**c**) side view of the detector.

The terahertz optomechanical detector proposed in this paper generally consists of three parts: the bi-material micro-cantilevers, metasurface absorber and the thermal insulation pillars. As depicted in Figure 1a, the metasurface absorber is employed to couple incident terahertz waves and placed between a pair of micro-cantilevers. Two bi-material microcantilevers with the same structure are arranged symmetrically beside the metasurface, specifically, their free ends (yellow frames in Figure 1a are connected with the metasurface), and their fixed ends (green frames in Figure 1a are connected with the thermal insulation pillars). The pillars are placed on silicon substrate to suspend the metasurface and micro-cantilevers, and to further realize thermal insulation between detectors and substrate. The top view and side view are also plotted in Figure 1b,c to show the symmetrical structure. While receiving terahertz waves, the free end produces a displacement corresponding to the incident power, so that the detector can obtain an optomechanical responsivity to characterize the incidence. In the following sections, the design, mechanism and performance analysis of bi-material micro-cantilever, metasurface absorber and the overall detector will be presented in turn.

### 2.1. Bi-Material Cantilever

**Figure 2:**
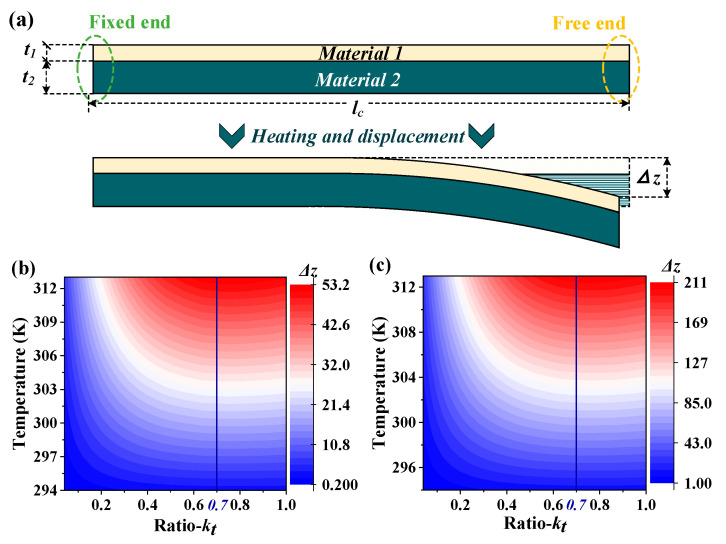
(**a**) Side view of bi-material micro-cantilevers; the length is *l_c_*, and *t*_1_ and *t*_2_ are the thicknesses of two materials, respectively; Δ*z* is the free end displacement. (**b**,**c**) The simulated thermomechanical responsivity of bi-material micro-cantilevers with different thickness ratios; the length of micro-cantilevers in (**b**,**c**) are 100 μm and 200 μm, respectively.

For terahertz detectors, the key indicators that greatly affect their performance include responsivity, response time and noise level. In this paper, the free-end displacement (denoted as Δ*z*) caused by a unit power terahertz incidence is employed to represent the responsivity (denoted as *R*) of the terahertz optomechanical detector. According to the thermal balance equation, the responsivity can be expressed as [28]:

(1) $$R=\frac{∆z}{P_{in}}=\frac{\eta }{G\sqrt{{1+\omega }^{2}\tau^{2}}}\;\frac{∆z}{∆T}=\frac{{\eta R}_{m}}{G\sqrt{{1+\omega }^{2}\tau^{2}}}\;,$$

where $P_{in}$ is the power of terahertz waves irradiated on a single detector; Δ*z* is the corresponding free-end displacement, as depicted in Figure 2a; *η* is the absorptivity of the detector for terahertz waves, which is mainly determined by the metasurface absorber; *G* is the thermal conductivity of detector; *ω* is the modulation frequency of the incident terahertz wave; *τ* refers to the thermal time constant, i.e., the response time of detector; and Δ*T* is the temperature change caused by incidence. The thermomechanical responsivity *R_m_* can be expressed as Δ*z/*Δ*T*. As displayed in Equation (1), the responsivity of the detector is positively related to its absorptivity and thermomechanical responsivity. In this section, the focus is concentrated to promote thermomechanical responsivity by optimizing bi-material micro-cantilevers.

The thermomechanical responsivity of bi-material cantilevers is first analyzed by Timoshenko [29], and subsequently refined by other researchers [30,31]. For the case where the displacement of the free end is much smaller than the length of micro-cantilever, the thermomechanical responsivity of bi-material micro-cantilever can be expressed as [29]:

(2) $$\frac{∆z}{∆T}=3\left(\alpha_{1}-\alpha_{2}\right)\frac{l_{c}^{2}}{t_{1}+t_{2}}{\left(1+\frac{t_{1}}{t_{2}}\right)}^{2}{\left[3{\left(1+\frac{t_{1}}{t_{2}}\right)}^{2}+\left(1+\frac{t_{1}}{t_{2}}\frac{E_{1}}{E_{2}}\right)\left(\frac{t_{1}^{2}}{t_{2}^{1}}+\frac{t_{2}}{t_{1}}\frac{E_{2}}{E_{1}}\right)\right]}^{-1}\;,$$

where *l_c_* is the length of the bi-material cantilevers, *α*, *t* and *E* are the thermal expansion coefficients, thicknesses and Young’s modulus of materials, respectively. The subscripts 1 and 2 represent two different materials employed in micro-cantilevers, respectively. According to Equation (2), the difference between thermal expansion coefficients of the two materials should be as much as it can be to obtain higher thermomechanical responsivity. Gold, silicon nitride and silicon substrate are adopted in this work due to their fabrication compatibility; the properties of these materials are listed in Table 1. In addition, the thickness ratio (expressed as *k_t_ = t*_1_*/t*_2_) between the two materials also plays an important role in the thermomechanical responsivity, specifically, the high order terms shown in Equation (2) demonstrate that the ratio has a complex and nonlinear effect on the responsivity. Multi-physics simulations are used to seek the optimal thickness ratio, in which two cantilever lengths of 100 μm and 200 μm are adopted, and the Si_3_N_4_ layer thickness is set to 500 nm and the gold layer thickness is set as a variable (*t*_1_). The boundary conditions are set to be a room-temperature (293.15 K) environment at one atmosphere. The bi-material micro-cantilever is heated by a temperature boundary which is set at the free-end interface, and another temperature boundary of 293.15 K is set at the fixed end to serve as the thermal insulation structure. Maintaining materials and other dimensional parameters unchanged, the simulated thermomechanical responsivity of bi-material (gold-Si_3_N_4_) micro-cantilevers with different thickness ratios is plotted in Figure 2b,c. Clearly, at any temperature, the maximum displacement (Δ*z*) occurs at a thickness ratio of 0.7. For the same temperature and thickness ratio, the displacement of longer micro-cantilevers is about twice that of the shorter one, showing good agreement with the constraint displayed in Equation (2).

As displayed in Equation (2) and Figure 2b,c, a longer cantilever can produce higher thermomechanical responsivity while heated by a constant temperature source. However, the metasurface absorbers behave more like a constant power source in practice. A longer cantilever will increase its volume and cause a lower temperature increment. Furthermore, the effective photosensitive area of the detector will also be affected and finally decrease its optomechanical responsivity. The design process of the entire detector in Section 2.3 will analyze and solve this problem.

Moreover, Equation (2) shows that the bi-material micro-cantilever with a thinner thickness (*t*_1_ *+ t*_2_) can produce greater thermomechanical responsivity. According to requirements of semiconductor processes, structures in the same layer should be of the same material and have the same thickness. As depicted in Figure 1, the silicon nitride layer and gold layer of bi-material micro-cantilevers are arranged in the same layer as the dielectric layer and pattern layer of metasurface absorber, respectively. Since the dielectric layer thickness plays an important role in the absorptivity of metasurface absorber, the thickness of each layer in the detector is mainly determined by the design of the metasurface absorber, which will be presented in Section 2.2.

### 2.2. Metasurface Absorber

Metasurface is an artificial two-dimensional periodic structure composed of plenty sub-wavelength scatterers [32]. In this work, the absorptivity and photothermal responsivity of metasurface absorbers are the main concerns. The absorptivity of metasurface absorbers (denoted as A(ω)) can be expressed as:

(3) A(ω)=1 − R(ω)− T(ω), 

where R(ω) and T(ω) represent the reflectance and transmission, respectively. Generally, the metasurface absorber can be regarded as a 2-port network, and the scattering parameters can be further employed to calculate the absorptivity as $R\left(\omega \right)={\left|S_{11}\right|}^{2}$ and $T\left(\omega \right)={\left|S_{21}\right|}^{\;2}$. Therefore, the absorptivity of metasurface absorbers depends on the reflection coefficient *S*_11_ and transmission coefficient *S*_21_. To obtain perfect absorption performance, both *S*_11_ and *S*_21_ should be as close to zero as possible, which also means a perfect impedance matching between metasurface and free space. In this case, most electromagnetic energy is absorbed by the metasurface and converted into heat, satisfying the needs of thermal detectors. Furthermore, since narrow-band metasurface absorbers are difficult to fabricate accurately, a wideband metasurface absorber is necessary to offset the fabrication errors.

**Figure 3:**
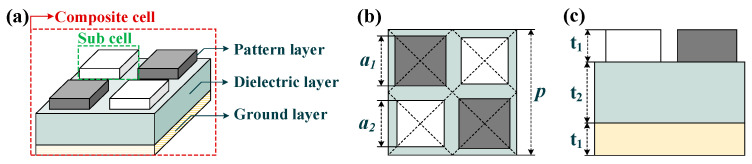
The (**a**) 3D view, (**b**) top view and (**c**) side view of a composite cell. The metal ground layer and pattern layer have the same thickness of *t*_1_; the dielectric layer thickness is *t*_2_. The period of composite cell is *p*; black and white sub cells have different side lengths, which are *a*_1_, *a*_2_, respectively.

As depicted in Figure 3, the metasurface absorber proposed in this paper consists of three layers from bottom to top, namely ground layer, dielectric layer and pattern layer. To maintain materials consistency with the bi-material cantilever, the dielectric layer is made from Si_3_N_4_, and the remaining two layers are gold. As a composite metasurface absorber, its composite cell consists of four sub cells in two different sizes. Specifically, the sub cells with a side length of *a*_1_, *a*_2_ are shown as gray and white colors, respectively. The sub cells are square patches with a resonance frequency inversely proportional to its side length. By adjusting their side length (*a*_1_, *a*_2_), two narrow absorption bands will merge together on the spectrum and thus form a single, wide absorption band. 

**Figure 4:**
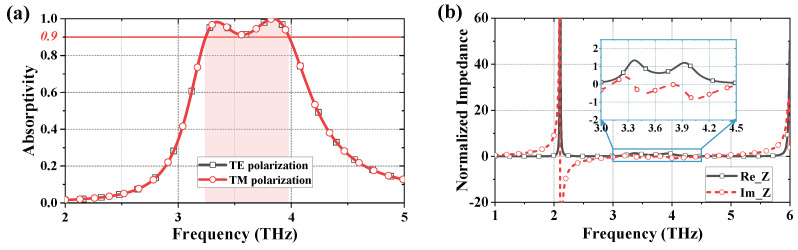
(**a**) The simulated absorption spectra of metasurface absorber under normal incidence with TE and TM polarization. (**b**) Normalized impedance of composite metasurface, and the magnified view within the absorption band (shown in blue frame).

With the optimized parameters listed in Table 2, the absorption spectra of metasurface absorbers are plotted in Figure 4a. An absorptivity higher than 90% is obtained from 3.24 to 3.98 THz, in which two absorption peaks higher than 98% occur at 3.35 THz and 3.84 THz, respectively. The normalized impedance of the metasurface absorber is extracted to explain the absorbing mechanism. Regarding the composite metasurface as uniform material and taking free space impedance as a reference, its normalized complex impedance can be extracted from the following equation [33,34]:

(4) $$\tilde{z}\left(\omega \right)=\sqrt{\frac{{\left(1+\tilde{r}\left(\omega \right)\right)}^{2}-\tilde{t}{\left(\omega \right)}^{2}}{{\left(1-\tilde{r}\left(\omega \right)\right)}^{2}-\tilde{t}{\left(\omega \right)}^{2}}},$$

where $\tilde{r}\left(\omega \right)$, $\tilde{t}\left(\omega \right)$ represent the complex reflection and transmission coefficient, respectively. As shown in Figure 4b, the real and imaginary parts of normalized impedance of the composite metasurface are plotted with a solid grey line and a dashed red line, respectively. To show the impedance matching in absorption band more clearly, the curves from 3.24 to 3.98 THz are zoomed in and re-plotted in the blue box. Note that the real part of normalized impedance fluctuates between 0.7 and 1.3 within the absorption band, and the imaginary part is limited between −0.6 and 0.4, which clearly illustrates that the composite metasurface has achieved good impedance matching with free space. As a result, most of the incident energy is absorbed rather than being reflected.

**Figure 5:**
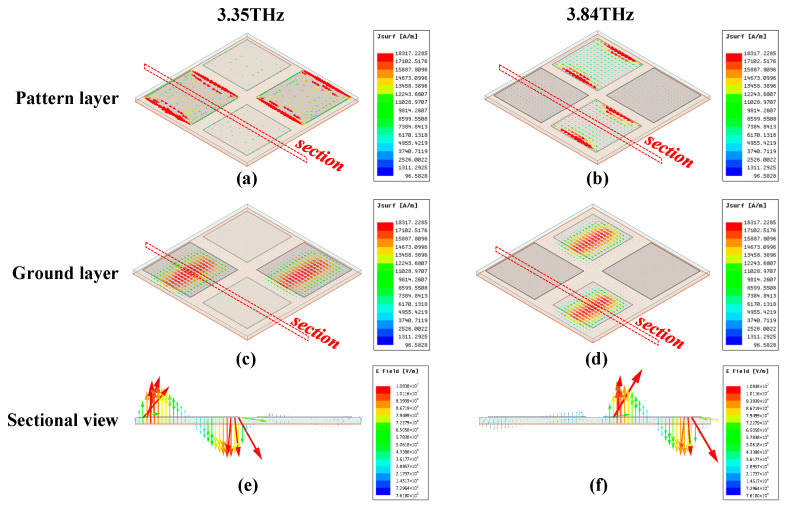
The surface current distributions in pattern layer at (**a**) 3.35 THz and (**b**) 3.84 THz, respectively. The surface current distributions in ground layer at (**c**) 3.35 THz and (**d**) 3.84 THz, respectively. The electric field vector in dielectric layer on the section at (**e**) 3.35 THz and (**f**) 3.84 THz, respectively.

To reveal the absorbing mechanism more intuitively, the surface current distributions on composite cell at these two peak frequencies are analyzed. As shown in Figure 5a,c, the surface current at 3.35 THz is mainly distributed on the larger-sized sub cells, corresponding to the gray patches shown in Figure 3; similar surface current distributions appear on the smaller patches at 3.84 THz. Moreover, as plotted in Figure 5a–d, the surface current distributions in the ground layer are antiparallel with that in the pattern layer for the same frequency. From the electric field in the dielectric layer as shown in Figure 5e,f, it is clear that several current loops are formed which behave like magnetic dipole resonances. As for the transmission, the ground layer thickness is much larger than the skin depth of terahertz waves in gold, so that terahertz waves cannot penetrate the metasurface and *S*_21_ can be regarded as zero. Therefore, the metasurface can realize wideband absorption with absorptivity higher than 90%.

**Figure 6:**
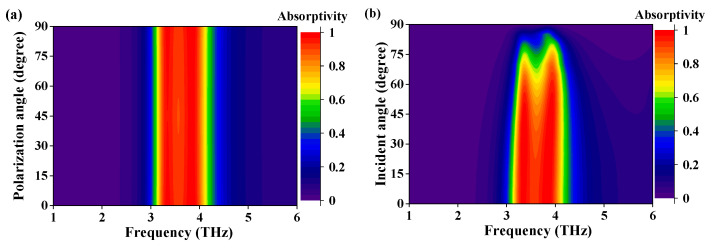
The simulated absorption spectra with (**a**) different polarization angles or (**b**) different incident angles.

The absorbing performances with different polarization angles and incident angles of metasurface absorber are also analyzed in this work. As plotted in Figure 6a, the absorption spectra with TE and TM polarized incident waves are perfectly consistent. However, as the signal coupling part of terahertz detectors, the absorbing characteristics for other polarization angles should also be taken into consideration. To obtain the absorption spectra with different polarized waves, the incidence is kept along the normal direction of metasurface and the polarization angle is set as a variable *φ* (TE polarization corresponds to *φ =* 0*°* and TM polarization is *φ =* 90*°*). Figure 6a indicates that for different polarized incidence, the absorption bandwidth can be maintained as stable, and the absorptivity in absorption band is higher than 90%. The absorbing characteristics for different incident angles are also analyzed while the polarization angle is kept at 0° (TE polarization). The angle between incident wave and normal direction of metasurface is set as variable *θ*. As shown in Figure 6b, the absorptivity in the band remains higher than 80% for *θ <* 45*°*. As for *θ >* 45*°*, the absorptivity and absorption bandwidth decrease rapidly. In conclusion, the metasurface absorber has performed satisfactory polarization insensitivity and incident angle stability.

**Figure 7:**
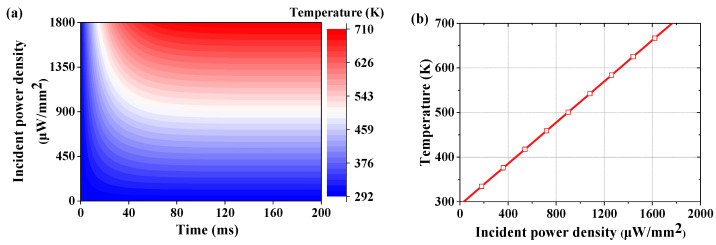
(**a**) The process of rising temperature. (**b**) The simulated photothermal responsivity of metasurface absorber.

Metasurface absorbers for terahertz detectors are also required to have significant photothermal responsivity. Since the metasurface proposed in this paper has a thickness of only 1.7 μm (0.02 λ), higher photothermal responsivity is expected. Multi-physics simulations are carried out to analyze the photothermal responsivity. As shown in Figure 7a, the temperature of the metasurface absorber reaches a steady-state value at 80 ms. To demonstrate the photothermal responsivity of the metasurface more clearly, the relationship between steady-state temperature and the incident power density is plotted in Figure 7b, which shows that there is a linear correlation between these two. The photothermal responsivity is calculated to be 0.23 K mm^2^/μW. Compared with the other absorbers, the metasurface absorber proposed in this paper achieves remarkable absorption performance and photothermal responsivity, which meets the requirements of optomechanical detectors.

### 2.3. Optomechanical Detector

The optomechanical detector is constructed on the basis of the micro-cantilevers and the metasurface absorber mentioned above. Consistent with the absorption band of the metasurface absorber, the operating band of the detector ranges from 3.24 to 3.97 THz. As depicted in Figure 1, the metasurface absorber is suspended by the micro-cantilevers arranged on both sides. To calculate the photosensitive coefficient of the detector, which is the ratio between absorber area and detector area, we define the detector area (*S_pix_*) as a rectangular area including air gaps, pillars, micro-cantilevers and metasurface absorber, as shown by the solid red box in Figure 1b; the absorber area (*S_ms_*) here refers to the area of the metasurface absorber, as shown by the dashed red box in Figure 1b. Therefore, the photosensitive coefficient is expressed as:

(5) $$A_{d}=\frac{S_{ms}}{S_{pix}}=\frac{{{(l}_{m})}^{2}}{\left(l_{p}+l_{c}+∆w\right)\times \left(∆l+2\times l_{p}\right)}\;.$$

**Figure 8:**
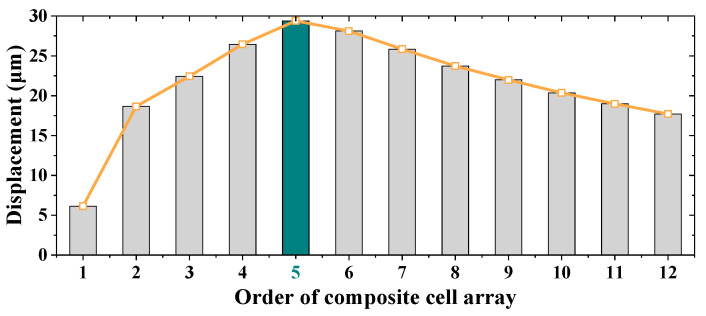
Displacement of the detector with different order of composite cell array.

The workflow of optomechanical detectors can be divided into two parts: namely the “electromagnetic energy-heat” conversion (called photothermal responsivity) achieved by the metasurface absorber and the “heat-displacement” responsivity (called thermomechanical responsivity) realized by the micro-cantilevers. As illustrated in Section 2.1, on one hand, longer micro-cantilevers can produce larger thermomechanical responsivity, but it will greatly decrease the photosensitive coefficient of the detector and consequently lower the sensitivity. On the other hand, enlarging the absorber area can increase the photosensitive coefficient, but photothermal responsivity of the detector will be decreased at the same time since more electromagnetic energy will be required to drive a single detector. To simplify this problem, we set the length of the bi-material micro-cantilever (*l_c_*) to be 10 μm larger than the side length of metasurface absorber (*l_m_*) in simulation. Since the metasurface absorber is a square array composed of several composite cells, the value of *l_m_* is an integer multiple of the composite cell side length. Keeping the boundary settings and the other dimensional parameters constant, the responsivity of detector to 1 μW terahertz radiation at 3.4 THz (the operating frequency of our terahertz Quantum Cascade Laser source which is employed in the measurement) is obtained by multi-physics simulations. As plotted in Figure 8, the largest displacement (29.4 μm) occurs while the metasurface absorber is composed of a 5 × 5 composite cell array. Therefore, the array scale of metasurface absorber is set to 5 × 5 and the optimized parameters of the detector are listed in Table 3. Furthermore, the sensitivity of the optomechanical detector is calculated to be 29.4 μm/μW, which means 29.4 μm free end displacement for per unit μW incidence.

Since the detector is covered with gold and Si_3_N_4_ which have low emissivity, radiative heat dissipation is negligible. The heat dissipation is mainly caused by the Si_3_N_4_ layer in micro-cantilevers, and the silicon pillars which is set as heat insulators. The total heat capacity (denoted as *C*) of the detector can be expressed as:

(6) $${C=\rho VC}_{det}\;,\;$$

where ρ, *V*, and *C_det_* are the density, volume and heat capacity of specific structures, respectively. Total heat capacity of the detector is calculated by adding the heat capacity of silicon pillars and Si_3_N_4_ micro-cantilevers. 

In the optomechanical detector, the metasurface absorber is regarded as the heat source, so the thermal conductivity of micro-cantilevers and pillars are the main concerns. Since the thermal conductivity of gold is much larger than that of dielectric materials, the gold cantilevers can be considered as a thermal short circuit. Therefore, the total thermal conductivity of the detector depends on that of the silicon pillars and the Si_3_N_4_ cantilevers, which can be expressed as:

(7) $$G=2\times \frac{A_{i}g_{i}}{L_{i}}\;,\;$$

where *A_i_* refers to the interface area between Si_3_N_4_ layers and silicon pillars, *g_i_* is the thermal conductivity of corresponding material, and *L_i_* the length of thermal insulation structure. The thermal time constant (expressed as *τ* = *C*/*G*) of the optomechanical detector is determined by its total heat capacity and thermal conductivity, which is calculated to be about 73 ms according to Equations (6) and (7).

The noise equivalent power (NEP) also plays an important role in the performance of detectors. Generally, there are three noise sources in thermal detectors: the noise caused by the continuous fluctuation of detector temperature (*NEP_TD_*), the noise caused by environment temperature fluctuation (*NEP_TB_*), and the thermomechanical noise due to thermally driven random motion of the mechanical structure (*NEP_TM_*). These three and the total noise (*NEP_T_*) can be expressed as [28,30]:

(8) $$\begin{array}{c} {NEP}_{TD}=T_{D}\sqrt{{4k}_{B}BG}/\eta \;,\\ {NEP}_{TB}=\sqrt{{16k}_{B}S_{pix}{B\sigma }_{T}\left(T_{B}^{5}+T_{D}^{5}\right)}/\sqrt{\left(\eta \right)}\;,\\ {NEP}_{TM}=\sqrt{{4k}_{B}{BT}_{D}/{Qk\omega }_{0}}/R\;,\\ {NEP}_{T}=\sqrt{{NEP}_{TD}^{2}+{NEP}_{TB}^{2}+{NEP}_{TM}^{2}}\;, \end{array}$$

where *T_D_* and *T_B_* are the temperature of detector and environment, respectively; *k_B_* and $\sigma_{T}$ are the Boltzmann constant and the Stefan–Boltzmann constant, respectively; *B* refers to the bandwidth of measurement system; *S_pix_* is the detector area, as depicted by the red frame in Figure 1b; *Q* is the quality factor of the detector, which is generally 1000 at room temperature and standard atmospheric pressure [28]; and *k* and $\omega_{0}$ are the spring constant and mechanical frequency of the detector, respectively, which are calculated by multi-physics simulations to be 0.011 N/m and 1463 Hz. The total NEP at 3.4 THz is thus calculated as:

(9) $$\begin{array}{c} NEP_{T}=\sqrt{NEP_{TD}^{2}+NEP_{TB}^{2}+NEP_{TM}^{2}}\\ =\sqrt{{\left(1.71\times 10^{-11}\right)}^{2}+{\left(3.8\times 10^{-13}\right)}^{2}+{\left(3.41\times 10^{-11}\right)}^{2}}\\ =3.82\times 10^{-11}\;W/\sqrt{Hz} \end{array}$$

Furthermore, the *NEP_T_* of the detector is equivalent to a displacement of 1.12 μm.

**Figure 9:**
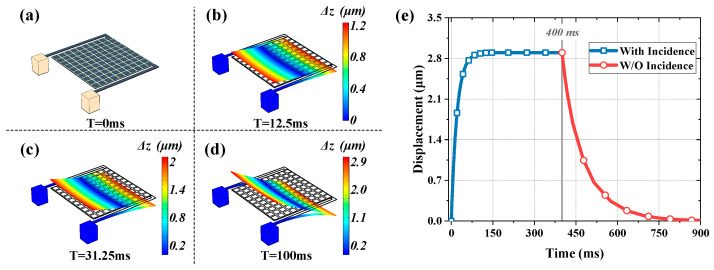
The simulated response process of detector under 100 nW incidence at 3.4 THz. Parts (**a**–**d**) corresponding to the deformation states of detector after irradiation of terahertz wave at 0 ms, 12.5 ms, 31.25 ms and 100 ms, respectively. Part (**e**) is the displacement of detector that changes over time.

Multiphysics simulations are taken to illustrate the optomechanical responsivity more intuitively and validate theoretical calculations, where the environment is set to room temperature (293.15 K) with one atmosphere, and the incident wave is 100 nW at 3.4 THz. The thermal deformation of 0 ms, 12.5 ms, 31.25 ms and 100 ms are shown in Figure 9a–d; moreover, curve of displacement vs. time of this process is also plotted in Figure 9e. It can be seen that the steady-state displacement occurs around 100 ms, which can be considered as the response time of the detector, as it is close to the calculated value (73 ms) as mentioned above. Furthermore, when the radiation source is removed at 400 ms, the recovery process takes approximately 250 ms.

## 3. Fabrication and Measurements

**Figure 10:**
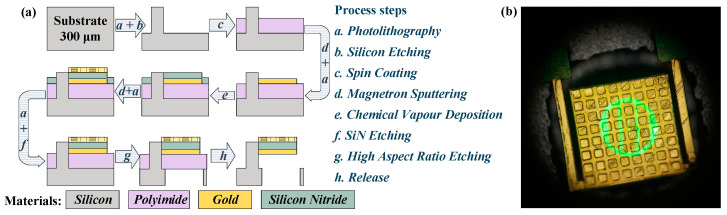
(**a**) The fabrication processes of optomechanical detector. (**b**) The top view of a single detector taken through a microscope.

The optomechanical detector proposed in this paper is fabricated on a silicon substrate. Since silicon substrate is opaque for visible light, and square aperture under detector is introduced for optical read-out. The fabrication processes are depicted in Figure 10a. Firstly, a silicon substrate with 300 μm thickness is etched to obtain silicon pillars; secondly, polyimide is spin coated as the sacrificial layer and a 0.5 μm gold layer is then prepared on the polyimide by magnetron sputtering technology to obtain the ground layer of metasurface absorber; thirdly, CVD and photolithography are employed to fabricate the 0.7 μm Si_3_N_4_ layer, which is the dielectric layer in the metasurface and bi-material micro-cantilevers; fourthly, another 0.5 μm gold layer is prepared on Si_3_N_4_ layer by magnetron sputtering and photolithography technologies to obtain the pattern layer of the metasurface and the gold layer of bi-material micro-cantilevers; and finally, high aspect ratio etching is used to get the optical apertures under each detector, and detectors are completely released by the isotropic dry etching of the polyimide sacrificial layer. The top view of a single detector taken through a microscope is shown in Figure 10b.

**Figure 11:**
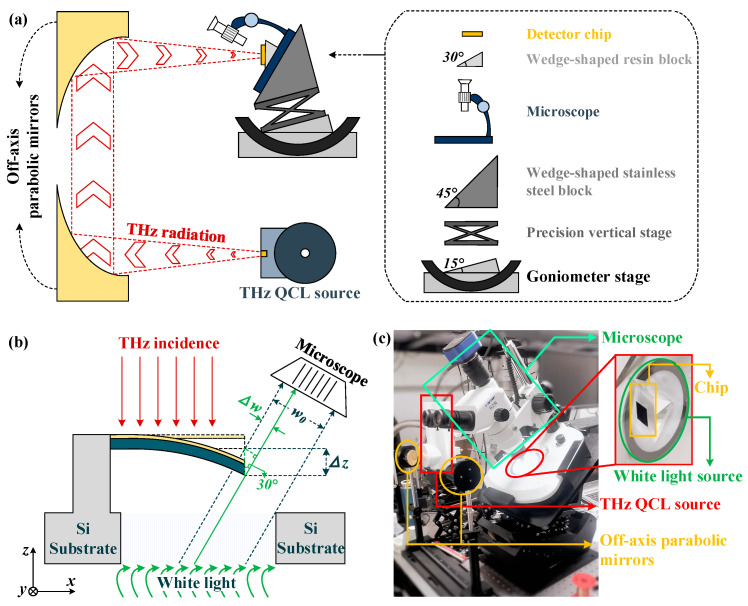
(**a**) Schematic diagram of the experimental system. (**b**) Schematic diagram of the relationship between optical slit width and detector displacement response. (**c**) The photo of experimental system, and the magnified view of detector chip.

To measure the responsivity of the optomechanical detector, the experimental system shown in Figure 11a is employed, including a terahertz QCL (Quantum Cascade Laser) source with 40 mW peak emission power at 3.4 THz, a pair of identical off-axis parabolic mirrors, an optical microscope, some platforms and precision stages. In order to ensure that the terahertz wave is vertically incident on the detector chip, the supporting structures of microscope and detector chip have been adjusted. As shown by the dashed box in Figure 11a, the chip is attached to a transparent wedge-shaped resin block, which is placed on the white light source of the microscope. The white light will pass the chip substrate through optical apertures under each detector, and after being blocked by the metasurface absorber in each detector, it will be observed by microscope as slits. Figure 11b illustrates that the terahertz incidence will cause the metasurface to move downwards, thereby changing the width of the optical-slit. According to trigonometric functions, the relationship between the detector displacement response (∆z) and the optical-slit width change (∆w) can be expressed as ∆z=∆w/cos60°. A photo of the experimental system is shown in Figure 11c, with the main devices marked. For clarity, the magnified view of the detector chip attached to a resin block is depicted in Figure 11c.

**Figure 12:**
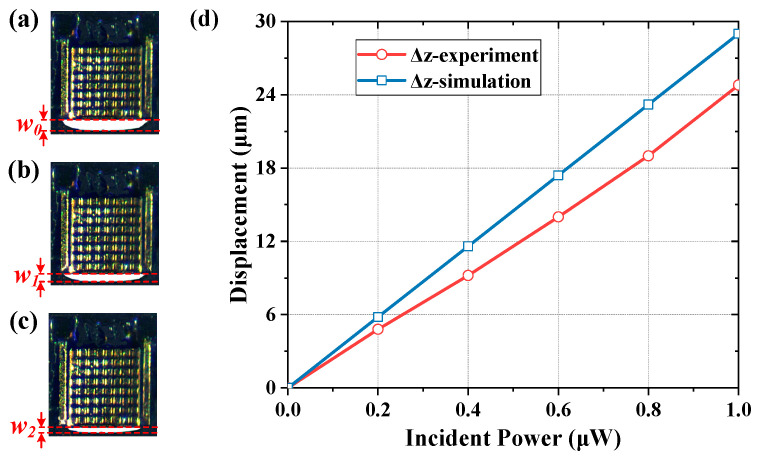
The measured response of the detector. Part (**a**–**c**) corresponding to incident power of 0 μW, 0.6 μW and 1 μW, respectively, and part (**d**) is the displacement response of simulation (blue line) and experiment (red line).

The terahertz QCL source can be regarded as a point radiation source, and two identical off-axis parabolic mirrors are employed to focus the terahertz energy on the detector. By placing the terahertz QCL source and detector chip at the focuses of two off-axis parabolic mirrors, respectively, most of the energy from terahertz QCL source can be harvested on the detector chip. The energy mainly loses are in the hemispherical space with the terahertz QCL source as its center. To reduce the loss of terahertz energy, the off-axis parabolic mirror with a short focal length (48 mm) has been used. During the experiment, the terahertz power irradiated on a single detector is tuned by changing the bias voltage of terahertz QCL source. When the power is 0 μW, 0.6 μW and 1 μW, the observed images are shown in Figure 12a–c, respectively. For comparison, the measured optomechanical responsivity and the simulated one are plotted in Figure 12d by red and blue lines, respectively. The measured optomechanical responsivity is calculated to be 24.8 μm/μW, which is lower than the simulated one. The difference can be explained by fabrication imperfections, specifically, the width of micro-cantilevers (*w_c_*) is made to be a bit larger, and there is a slight deviation in alignment for different layers of the detector.

## 4. Conclusions

In summary, this paper has demonstrated the design, analysis, fabrication and measurements of an optomechanical terahertz detector. A metasurface absorber with absorptivity higher than 90% from 3.24 to 3.98 THz is employed to convert incident electromagnetic energy into heat, and a pair of bi-material micro-cantilevers are symmetrically arranged on both sides of the metasurface to produce significant displacement. Silicon pillars are introduced as the thermal insulators. The detector is fabricated on a silicon substrate with a thickness of 300 μm, and several optical apertures on the substrate under metasurface absorber are prepared for optical read-out. To measure the optomechanical responsivity of this detector, an optical read-out system based on terahertz QCL source and off-axes parabolic mirrors is developed. The measured optomechanical responsivity is 24.8 μm/μW, showing good agreement with the simulated one. Images observed by optical read-out system indicate that the detector has potential to be further developed for use in focal plane arrays, realizing real-time terahertz imaging at room temperature. 

## Figures and Tables

**Table 1:** Mechanical, thermal and electromagnetic properties of materials.

Material	Thermal Expansion Coefficient *α* (×10^−6^ K^−1^)	Thermal Conductivity *g* (Wm^−1^ K^−1^)	Heat Capacity *c* (Jkg^−1^ K^−1^)	Young’s Modulus *E* (×10^9^ Pa)	Density *ρ* (×10^−3^ kg/m^3^)	Electric Conductivity *σ* (×10^6^ Sm^−1^)	Relative Permittivity *ε_r_*
Si	2.7	131	748	100	2300	-	11.8
Si_3_N_4_	2.3	20	700	250	3100	-	7.5
Au	14.8	317	129	70	19,300	45.6	-

**Table 2:** The optimized parameters of metasurface absorber.

Parameter	*a*_1_	*a*_2_	*p*	*t*_1_	*t*_2_
**Value (μm)**	13.8	15.2	18.5	0.5	0.7

**Table 3:** The optimized parameters of detector.

Parameter	*l_m_*	*l_c_*	*l_p_*	*l_h_*	**∆*l***	**∆*w***	*w_c_*	*w_h_*	*t_p_*	*t_sub_*
**Value (μm)**	185	195	30	220	180	15	8	200	30	270

## Data Availability

The data presented in this study are available from the corresponding author upon reasonable request.
